# Could Vhavenda initiation schools be a panacea for HIV and AIDS management in the Vhembe district of South Africa?

**DOI:** 10.4102/curationis.v45i1.2356

**Published:** 2022-11-30

**Authors:** Avhatakali A. Ndou-Mammbona, Azwihangwisi H. Mavhandu-Mudzusi

**Affiliations:** 1 Department of Health Studies, University of South Africa, Pretoria, South Africa

**Keywords:** Ethnography, HIV infections, traditional healers, traditional initiation schools, Vhavenda

## Abstract

**Background:**

Several human immunodeficiency virus (HIV) and acquired immunodeficiency syndrome (AIDS) prevention strategies have been implemented to counteract the impact of the disease, including the use of condoms, social marketing, testing, voluntary counselling and education programmes. One of the platforms which has not been fully explored is that of traditional initiation schools.

**Objectives:**

This study aimed to explore and discuss Vhavenda traditional initiation schools, which can be used as panacea for HIV and AIDS management in the Vhembe district of South Africa.

**Method:**

This ethnographic study was conducted in the Vhembe district of South Africa, among nine purposively sampled key informants drawn from a cohort of Vhavenda traditional healers and leaders. Data were collected using semistructured face-to-face interviews and analysed using ethnographic content analysis.

**Results:**

The results indicate that Vhavenda traditional initiation schools positively affect the management of HIV and AIDS. Initiation schools are centres for cultural education and the formation of a cultural identity. During the initiation process, initiates are taught social norms, customs and values which will serve them well in adulthood. They are also taught matters of sexuality, courtship, marriage and respect for others.

**Conclusion:**

The positive attributes of Vhavenda initiation schools should be accommodated and implemented in curricula from the primary school level up to the tertiary level to reduce and curb the spread of HIV infection.

**Contribution:**

Improved expertise at the initiation schools will aid the Department of Health and Education and Training to develop and implement suitable cultural contextualised HIV and AIDS prevention strategies.

## Introduction

The impact of human immunodeficiency virus (HIV) and acquired immunodeficiency syndrome (AIDS) on communities remains a significant concern worldwide. New HIV cases in the Vhembe district, Limpopo province are reported to be at 64 372 – approximately 11.4% of the total population (District Health Information System [DHIS] 2020:13). This is lower than the HIV incidence in Capricorn district (70 710, approximately 19.4% of the total population) (DHIS 2020:08), where the high incidence rate may be because of the urban nature of the district, which is home to fewer traditional initiation schools than Vhembe. Several platforms are used to enhance these strategies, and community facilities such as schools and clinics are used to educate people about HIV prevention.

Attendance of an initiation school is an African custom that marks the transition to another stage of life in traditional communities (Emberly & Tshitokisi 2020:110). Such schools differ from one culture and ethnic group to the next. For the Batsonga, Vhavenda and Varemba people of Zimbabwe, initiation for both boys and girls not only marks a period in life when they transition into adulthood but also a period of societal approval and acknowledgement (Mapindani 2018:84). Notably, the rites of passage differ according to gender, in most instances. Where young men are initiated in preparation for adulthood, the rites of passage are accompanied by the societal duties associated with being a man (Mohlaloka, Jacobs & De Wet 2016:22). This takes place not only through circumcision: initiates are also taught a sense of responsibility, respect, value for life and resoluteness (Douglas & Maluleke 2018:587).

Admittedly, the process is mainly signified through male circumcision, which is a very important aspect (rite) of the entire ritual. In most African countries, the aim of initiation schools is to circumcise and prepare young boys for their conversion to ‘manhood’ (maturity), in keeping with a cultural philosophy (Prusente, Khuzwayo & Sikweyiya 2019:02). Young men are educated about courtship and marriage, cultural expectations of social responsibility and male behaviour in society. As a result, in the community, men are endowed with many privileges associated with their state of maturity after initiation (Prusente et al. 2019:03).

In the context of initiation, circumcision surgery is performed by a traditional doctor. The practice has garnered widespread attention over the past decade because of the many botched circumcisions and deaths of young initiates. Uncertainties remain about conventional surgical processes that can increase HIV transmission and about the viable impact of cultural guidance on sexual risk-taking and gender-based violence, which are also major drivers of HIV transmission. Improved expertise at these intersections is important for developing and implementing suitable HIV prevention strategies (Prusente et al. 2019:04).

Girls also attend initiation school as a rite of passage into womanhood. The scope of knowledge covered includes life skills and how the female body functions (Tshifhumulo 2022:343). Although some information is available about such schools, there is a need to thoroughly explore and discuss the possibility of using initiation schools to address the problem of HIV and AIDS prevalence in the Vhembe district of South Africa.

## Methods

### Design

The researchers utilised an ethnographic design, as it captures the social meanings and ordinary activities of members of the community in their natural settings or contexts (Brink, Van der Walt & Van Rensburg 2018:106). As Vannini (2018:04) elaborated, ethnographic design is the in-depth study of a people’s way of life, of their culture. Ethnographic design enabled the researchers to capture certain cultural activities of the Vhavenda people which may influence their HIV management, with a view to critically analysing and interpreting Vhavenda traditional initiation practices in relation to HIV and AIDS management.

### Study setting

The study was conducted in one of the rural districts of Limpopo province. The Vhavenda people of the Vhembe district are culturally rooted and still engage in traditional initiation schools.

### Population and sampling

The study population comprised all the traditional leaders and healers involved in initiation schools in Vhembe district. Purposive and snowball sampling were used to select participants in the study. Because of the ethnographic nature of the study, purposive sampling was first used, followed by snowball sampling. The researchers first approached a traditional leader who is an acknowledged expert (purposive sampling) (Polit & Beck 2017:254). The traditional healer is frequently interviewed on Phalaphala FM Radio, a Tshivenda station regarding Vhavenda cultural practices and rituals. The researchers were also referred to other individuals who were deemed experts in initiation schools and could serve as key informants (snowball sampling) (Gray, Grove & Sutherland 2017:345). The demographic data of the nine key informants involved in the study are presented in Table 1.

**TABLE 1:** Demographic data of the key informants.

Pseudonym	Age in years	Gender	Traditional practice	Years of practice
Ramabuli	82	Male	Traditional leader, initiation schools (*vhusha, domba* and *musevhetho*)	48
Muofhe	59	Female	Traditional healer, initiation school conductor (*vhusha, domba* and *musevhetho*)	27
Maitele	71	Male	Traditional healer, circumcision school (*murundu*)	31
Maano	75	Male	Traditional healer, circumcision school (*murundu*)	54
Ndiafhi	54	Male	Traditional healer, circumcision school (*murundu*)	42
Nyambeni	80	Male	Traditional healer, circumcision school (*murundu*)	49
Khedzi	70	Male	Traditional leader, initiation schools (*vhusha, domba* and *musevhetho)*	41
Masiagwala	73	Male	Traditional leader, initiation schools (*vhusha, domba* and *musevhetho*)	33
Razwinani	49	Male	Traditional leader, initiation schools (*vhusha* and *domba*)	28

Of the six traditional healers and three traditional leaders, eight were male and one was female. Their ages ranged from 49 to 82 years.

### Data collection

Prior to collecting the data, the researchers developed an interview guide comprising four questions (translated into Tshivenda, as the interviews were conducted in that language):

- What are the basic practices offered in the various traditional initiation schools for Vhavenda people in the Vhembe district?
- What are the main aims of these initiation schools?
- What is the information provided during the initiation schools?
- What are the potential implications of those practices for HIV management?

To guide the observation, the researchers developed an observation tool with questions focusing on the types of initiation schools, the processes followed, the individuals involved and the implications of those processes for HIV management. The interview guide and observation tool were initially tested by interviewing and observing a community elder while performing cultural practices related to *lobola* (paying of the bride-price), which are not part of the initiation process. The interview guide and observation tool were revised and retested by interviewing another traditional healer, performing the cultural practice of ‘*u thusa nwana*’, a process of protecting and fortifying an infant against illness and terror as the child prepares to appear in public for the first time (Mahwasane 2012:02).

The first author collected the data between August and December 2019. All the interviews, except for those with the individual initiating *murundu* (traditional initiation school for boys where they are circumcised), were conducted where the activities were taking place (*musevhethoni, vhushani* and *dombani*). For *murundu*, the challenge was that because the researcher is female, she was barred from visiting the school – a cultural taboo for women. The second obstacle was that *murundu* is conducted in winter, May 2022 – July 2022, which fell outside of the data-collection period. Data were collected in the form of individual semistructured interviews and observations (concurrent, where possible), following ethnographic design principles. Field notes were also taken to record nonverbal cues and other aspects which could not be recorded or captured via the observation tools. Probes and prompts were used to elicit more detailed information from the key informants. All interviews were audio-recorded, and each lasted approximately 45–60 min.

### Analysis of data

The audio-recorded interviews were transcribed verbatim and translated from Tshivenda into English. Transcripts, observation tools and field notes were analysed manually, using ethnographic content analysis (Brink et al. 2018:107; Bryman & Bell 2014:336). This process entailed the researchers and an independent coder individually reading all the transcripts, field notes and observation tools to code and categorise themes. Next, they searched for relationships across developing themes and developed a master table with superordinate themes, subthemes and quotes from the transcripts before comparing and discussing the data to produce a refined master table. Co-coding occurred independently to ensure conformability and reliability (Brink et al. 2018:159).

### Ethical considerations

The Department of Health Studies and the Research Ethics Committee of University of South Africa granted the researchers ethical clearance for this study. Permission was also obtained from the Department of Cooperative Governance, Human Settlements and Traditional Affairs under the Vhembe House of Traditional Leaders (Vhavenda Kingship Council). Throughout the study, ethical principles were observed by using pseudonyms to ensure the confidentiality and anonymity of the participants. Voluntary informed consent was obtained from all key informants. Sacred cultural practices were respected, and the researchers adhered to cultural requirements during data collection. Researchers were dressed in a culturally appropriate manner and adopted a seated position to show respect (sitting on an animal hide and speaking Tshivenda when conducting interviews). ‘Door and mouth openers’ (monies required when entering the homes of traditional leaders and healers) were paid to seek their permission to conduct research. The researchers documented only that information which they were permitted to use, as some details are regarded as sacred; for example, male initiation school camps are taboo for women to enter. No videos or photographs were taken while observing the events.

## Trustworthiness of the study

To ensure rigour in the study, the framework developed by Guba and Lincoln (1994) was adhered to. The criteria for ensuring trustworthiness were credibility, confirmability, dependability, transferability and authenticity (Polit & Beck 2017:559). A complete description of the setting and key informants was provided to ensure transferability, and to guarantee authenticity, interviews were audio-recorded and transcribed verbatim to capture each key informant’s voice. Each of the audiotape recordings were transcribed verbatim and analysed by both the researchers and an independent coder to ensure dependability. All key informants were given feedback on the emerging interpretations to garner their insights and ensure credibility. In constructing a single master table, the researchers and independent coder established the assumption of confirmability, without emphasising their own perspectives or biases.

## Results

Two superordinate themes emerged from the data analysis, namely aspects of Vhavenda traditional schools which may promote HIV and AIDS management and gaps identified at initiation schools that may fuel the spread of HIV. Under each superordinate themes, several subthemes emerged (see Table 2).

**TABLE 2:** Summary of results.

Superordinate themes	Themes	Subthemes
**1. Aspects of Vhavenda traditional schools which may promote HIV and AIDS management**	**1.1 *Promoting abstinence and faithfulness***	1.1.1 *Delayed sexual debut*
		1.1.2 *Early engagement for marriage, to prevent multiple concurrent partners*
		1.1.3 *Infertility management*
	**1.2 *Role clarifications for different stages of initiation schools***	1.2.1 *How to behave as a married man*
		1.2.2 *How to behave as a married woman*
		1.2.3 *Forms of address*
		1.2.4 *Preparation for sexual activity*
	**1.3 *Averting conflict between couples***	1.3.1 *How to behave towards the opposite sex*
		1.3.2 *Mediator’s role in a married couple*
		1.3.3 *Teaching mutual respect*
**2. Gaps identified at initiation schools that may fuel the spread of HIV**	**2.1 *Nonadherence to infection, prevention and control measures***	2.1.1 *Use of a single knife*
		2.1.2 *One traditional surgeon making the incision on all initiates*
	***2.2 Gender discrimination***	2.2.1 *Male dominance at all traditional schools*
		2.2.2 *Reduced libido among women*
		2.2.3 *Emphasising tolerance*

HIV, human immunodeficiency virus; AIDS, acquired immunodeficiency syndrome.

### Aspects of Vhavenda traditional schools which may promote HIV and AIDS management

In focusing on those aspects of Vhavenda traditional schools which may promote HIV and AIDS management, the findings were categorised according to the following themes: promoting abstinence and faithfulness, role clarifications for different stages of initiation schools and averting conflict between couples.

#### Promoting abstinence and faithfulness

Boys and girls are encouraged to remain virgins until marriage and to be faithful in their marriages. This is done to curb promiscuity and reduce the risk of being infected with HIV.

**Delayed sexual debut:** Rewarding virginity is deemed to delay girls from engaging in sexual intercourse. Boys attend female initiation schools, form friendships, dance *tshinzherere*, learn how to sit, how to respond and greet and how to play together harmoniously up to 18 years old, without engaging in sexual activity. It is also believed that restricting a high protein diet for girls and boys slows the onset of physical maturity, which delays sexual awakening:

‘In all-female initiation schools, virginity testing is conducted to ward away [*i.e. prevent*] females from engaging in sexual intercourse before marriage. The girls will be afraid to have sexual intercourse, as this will be revealed during initiation. The main purpose of doing the above is to reduce HIV incidence and its spread. Girls will be afraid of engaging in sexual intercourse to avoid embarrassment for themselves and the family. It also reduces one’s chance of being married to a well-off family, as the girl will no longer [*be*] regarded as pure.’ (Muofhe, 59 years old, female)

Maano concurred that *musevhetho* plays an important role in the management of HIV, as the teachings given to initiates inhibit them from engaging in premarital sex:

‘At *musevhethoni*, girls are taught not to engage in sexual intercourse before marriage. They are encouraged to remain virgins so that they will not get infected with HIV [*and*] AIDS in the process. It is an insult to the family and shame on yourself to be given a broken calabash, to symbolise that you are no longer a virgin before marriage.’ (Maano, 75 years old, male)

Diet is important in delaying sexual debut for both males and females, as this participant indicated:

‘We feed the initiates with only stiff pap without any soup or relish, until the wounds heal. This is to avoid the use of any relish which may stimulate penile erection before the wound heals. If one engages in sexual intercourse before healing, it predisposes one to contract HIV. Diet is also important, as it reduces the chance of one becoming obese and ending up with diseases like sugar diabetes and high blood pressure.’ (Nyambeni, 80 years old, male)

**Early engagement for marriage, to prevent multiple concurrent partners:** Older girls and boys are advised to engage in sexual intercourse only after marriage, to avoid taboos (*matula*) such as getting pregnant and having babies:

‘At *murunduni* and *mesevhethoni*, both girls and children are taught to remain virgins as early as possible to avoid *matula* [*taboos*] and infections like HIV [*and*] AIDS. If we delay, we will never tame them.’ (Razwinani, 49 years old, male)

**Infertility management:** A traditional healer is employed to treat infertility at *thondoni* for older boys who are ready to marry. This is to prevent them from engaging in extramarital relationships in their quest for a child – an activity which may predispose both man and wife to HIV infection. The main purpose is not only to screen boys but also to treat the problem:

‘We send older boys who are ready for marriage at *thondoni* to the traditional healer if it was discovered that they cannot father a child. They are encouraged to masturbate early in the morning, after that they release their sperm inside [*a*] small calabash with water to see if the sperm they produce is fertile; floating sperm symbolises that the man is infertile, and sperm sitting underwater is a symbol of fertility.’ (Ndiafhi, 54 years old, male)

A boy deemed unable to father a child is sent to the traditional healer for treatment:

‘The man is sent to the traditional healer known by the elders, who will then treat the man, and the man will be able to father a child.’ (Masiagwala, 73 years old, male)

#### Role clarifications for different stages of initiation schools

A mediator helps with role clarification during initiation schools, where boys and girls are taught how to conduct themselves in society.

**How to behave as a married man:** Men are taught how to take care of a woman and are advised on how to become good fathers. One participant is of the view that a husband need to know when the wife is in her menstrual period, in order to avoid sleeping together. This is done to minimise chances of transmitting HIV, if one of them is living with HIV:

‘At *thondoni*, we teach older boys who are ready [*for*] marriage to know womanhood, that when their women are menstruating, they must not have sexual intercourse with them for seven days. This is done to prevent sexually transmitted infections, as they will be in contact with raw blood.’ (Maano, 75 years old, male)

**How to behave as a married woman:** In Vhavenda tradition, a married woman must behave in an acceptable manner. She is not supposed to engage in sexual intercourse with other men; she must show respect – for instance, when offering food to her husband, she must kneel. As one participant explained, a convivial marital set-up helps to prevent the man from having an extramarital affair, thereby indirectly countering promiscuity and the possible spread of HIV:

‘A woman is taught to behave well and not go around sleeping with other men as if she is selling herself. The reason being that she must not contract HIV and end up spreading it to others and her husband.’ (Masiagwala, 73 years old, male)

Female initiates are taught how to take care of their babies and to wait for the father of the child to return if he is working far away, without giving in to the temptation of engaging in sexual relations with other men:

‘Girls are taught how to take care of a baby; it is in preparation for married life. She must know how to feed the baby. The woman must breastfeed the child up to two years. This is a form of prevention of unwanted pregnancy, as the woman will not engage in sexual intercourse if the husband is working far; she will be able to wait until he comes back. It is a way of preventing and reducing [*the*] child mortality rate. If the child has malnutrition and is living with HIV, it lessens the life span, as the child can die due to HIV [*and*] AIDS complications.’ (Muofhe, 59 years old, female)

**Forms of address:** In preparation for marriage, an older girl who has undergone traditional school should also go to *thondoni* for advice on how to be a good wife and how to behave, after which a new name is given to her, the main purpose being to show the whole community that she is married and thus unavailable. All the names given carry the prefix *Nya-* (e.g. *Nyamasindi* or *Nyamukumela*). Married women are supposed to wear *misisi* (Vhavenda traditional attire meant for older girls or married women) instead of wearing *masheḓo* (Vhavenda traditional attire that covers only their private parts and expose the buttocks and the side of the thighs) like an unmarried woman would, as a show of respect and to prevent other men from proposing to her or being intimate with her:

‘At *thondoni* school, older boys are told that when they marry, they should give their wives new names as tradition permit[*s*], to show others that the woman is married so that other men would not make advances on her. In turn, she also will not sleep with another man. Women are taught to dress appropriately as married women. Dressing differently when married also make[*s*] her be faithful and not engage in sexual relationships other than [*with*] her husband. This will also reduce the HIV incidence.’ (Muofhe, 59 years old, female)

**Preparation for sexual activity:** Older girls are advised at *thondoni* how to behave when married. They are instructed on how to satisfy a man sexually. A demonstration is given in the form of a song, which conveys a message on sexual activities to all initiates:

‘At *thondoni*, older boys and girls are taught on how to please a man (in case of a female) and how to please a woman (in case of a male) sexually. There is a song called *mabidigama*, where the mature male [*and*] female youth are given instructions about sex, that it is only done by married people.’ (Ramabuli, 82 years old, male)

#### Averting conflict between couples

The participants indicated that at initiation schools, initiates are taught conflict prevention strategies. This theme comprises the subthemes: how to behave towards the opposite sex, mediator’s role in a married couple and teaching mutual respect.

**How to behave towards the opposite sex:** Apart from being given rules, initiates are taught how to behave towards the opposite sex. At *murunduni*, men are taught not to engage in sexual intercourse when the woman has her period, as this increases their chance of becoming infected with HIV and other sexually transmitted infections (STIs) where one partner is already HIV positive. They are taught that, after day 7, they can engage in sexual activities with their wives, as the following statement indicates:

‘Men are taught not to sleep with their wives if the women are menstruating, as it increases their chance of being infected if one or both are already infected with HIV. They are advised to wait for seven days. After that, they can enjoy having sex with their wives again.’ (Khedzi, 70 years old, male)

**Mediator’s role in married couple:** An elder is assigned to each initiate, to offer advice in preparation of marriage. Vhavenda tradition is very rich in that, at initiation school, men are assigned a mediator whom they can consult if there is a problem at home. Many relationship-based problems may be solved by that elder:

‘During initiation schools, people are told to consult the mediator. Men are advised not to fight with their wives. They have delegated a mediator to consult if they have problems. Men are taught not to solve problems related to *vhudzekani* [*sex*] as they will end up having extra-marital affairs to quench their desire, resulting in [*them*] bringing sexually transmitted infections (including HIV) home, if their women are not submitting to it. The men will have to consult the elder regarding the problems [*they have*], to solve [*them*], then they will leave [*in*] peace.’ (Nyambeni, 80 years old, male)

**Teaching mutual respect:** Boys and girls are taught by their different initiation schools to respect one another and remain faithful to their partners. Although men are also advised to marry more than one wife, they are still expected to respect and be faithful to all their wives, to prevent contracting and/or spreading HIV:

‘You think we only expect girls to be faithful? It is not the case, also boys are taught to be faithful and respect their wife/wives, to avoid being infected with HIV. It is just that boys are boys, they know that what they are doing is wrong, and they are not supposed to cheat [*on*] their partners.’ (Maitele, 71 years old, male)

‘In *murunduni*, men are taught to respect their wives, they are taught that they must love their wives.’ (Khedzi, 70 years old, male)

### Gaps identified at initiation schools that may fuel the spread of HIV

#### Nonadherence to infection, prevention and control measures

The problem with some circumcision schools is that they do not comply with infection prevention and control practices. Another is that the environment in which the initiates are housed is not clean, because makeshift shelters made of plastic and cardboard boxes are used. Often, the instrument used by the traditional surgeon making the incisions is not clean or properly disinfected; methylated spirits are used as disinfectant. Some allege that the knife used is sanctified by the traditional healer and thus cannot spread infections to initiates:

‘We erect temporary house[*s*] in the bush for housing initiates. We also use bush[*es*] nearby as our toilets, but one should relieve himself far away. You cannot find anyone infected with HIV through initiation schools. I also use methylated spirit[*s*] in between the initiates while removing the foreskins.’ (Maitele, 71 years old, male)

#### Use of a single knife

Some initiation schools use one knife on all initiates, without observing infection and prevention control measures, thus increasing the chance of spreading HIV or other infections:

‘This whole process is sacred, so there is no way I can use other knives, because the knife I used is sanctified. It is not an ordinary knife. It can also dispel any kind of infections amongst the initiates. You would not understand how we, as initiators of initiation schools, operate. I assure you there is no way that a boy can get infected with HIV, because we are using one knife. The only thing done is to wash the knife under a concoction made by the traditional healer before it is used on another initiate for cutting of the foreskin.’ (Masiagwala, 73 years old, male)

**One traditional surgeon, making the incision on all initiates:** Since the incision is performed by one traditional surgeon, there is the possibility of him not washing his hands or changing gloves during multiple procedures. This may lead to the spread of HIV infection:

‘I am telling you [*Nga khaladzi nga Makwarela lupedzi lwa mutahabvu, “I can swear by sister Makwarela, who is the lastborn to my father”*] that no one can get infected with HIV due to our deed. I only use traditional medicine from our ancestors; I have been using it for many years without any problems. You can tell the government to come and do research to see if they can pick any initiates who [*are*] found to be infected with HIV after coming from initiation school.’ (Maano, 75 years old, male)

#### Gender discrimination

**Male dominance at all traditional schools:** During all the stages of the initiation school, male dominance is evident. Most schools are run by men – they are deemed to be the experts – and are a family business. Not just anyone can do it; women cannot conduct circumcisions, as that is regarded as taboo.

**Reduced libido among women:** Young women’s clitorises are burned at initiation schools, to decrease libido until they marry. As the participants indicated, this is performed to protect young women from becoming infected with STIs such as HIV because of early sexual activity:

‘We burn the clitoris with hot wire, to make a woman less sexually active. The aim is to ensure that the girl is not interested in sex with any available man. Therefore, they become good wife material, who remain faithful. Even if they are married, the man might even come home once per year, and the women will settle. The situation even makes some of the women to be married by women [*o ya u bikela Makhadzi*], and they will only have sex with a man just for procreation purposes, not for pleasure.’ (Muofhe, 59 years old, male)

**Emphasising tolerance:** The endurance that the women display when their clitoris is burned at initiation school results in them being viewed as tolerant. They are instructed to love their husbands, no matter what, and to remain faithful:

‘Women are told to stay with their husband until death. A woman is expected to endure all problems encountered at her husband’s house. There is a saying in Tshivenda which says the women’s place of burial is at her husband’s house.’ (Muofhe, 59 years old, male)

## Discussion

Among the nine key informants, only one was female. This gender disparity is an issue of concern, as four of the traditional practices (*musevhetho, vhusha, domb*a and *thondo*) are aimed at girls, although there is also *thondo* for boys. The issue is that men and boys are allowed to initiate and even participate in female-targeted initiation schools, while women and girls are not allowed in male initiation schools such as ‘*murundu* (traditional initiation school for boys)’. This sentiment was also shared by Mudau et al. (2018:190) when they stated that boys are more dominant in all female traditional initiation schools, whereas girls are prohibited from participating in *murundu*. The ages of the initiators ranged from 49 to 82, but notably, only three were younger than 60 (49, 54 and 59), while the majority were 70 years and older (70, 71, 73, 75, 80 and 82, respectively). Their age is a challenge, as it means only older generations are involved in traditional initiation schools. This may affect the continuity and sustainability of the practices, in cases where the older generation pass on without mentoring the younger generation. According to Mudau et al. (2018:191), children should be taught and encouraged to attend traditional initiation school as they are being guided and given rules in the process. This is also done so that the older generation can impart their knowledge and skills about *ulaya nwana* to the younger generation.

Traditional initiation schools have both a beneficial and a negative impact on HIV management. Some of the reported benefits include girls and boys being educated on how to comport themselves to avoid being infected with HIV (Mudau et al. 2018:181). Traditional values teach men to respect their wives in marriage and to comprehend the nature of women. This helps to avoid confrontations around the relationship and sexual intimacy. In the same way, girls attend initiation school as a rite of passage into adulthood, and the scope of knowledge covered includes life skills and human philosophy (Tshifhumulo 2022:342). Older boys are encouraged to respect, love and remain faithful to their partner(s) throughout their married life. They are not supposed to force women to sleep with them or to initiate sex if the woman is menstruating. As Mudau et al. (2018:184) noted, boys at initiation school are guided not to engage in sexual risk-taking and gender-based violence, both of which are major drivers of HIV transmission. It is believed that sexual assault leads to an increase in HIV infections. Girls and boys are taught about sexuality, marriage, courtship and respect in all stages of initiation schools. As Rathebe (2018:06) confirmed, the initiation school is where adolescent girls and boys go to learn about the values, beliefs, struggles, respect and accountability associated with their cultural tradition. This echoes the opinion of Morei (2017:07) that initiates are taught on matters of sexuality, courtship, marriage and respect for others, while conceding that rites of passage vary from one culture to another. Not only do they learn to respect one another and their culture’s ways of life at initiation schools, but both boys and girls are assigned mediators to whom they can speak if they have any problems. Both boys and girls are taught about their respective roles as husbands and wives. Douglas and Maluleke (2018:585) concurred that initiation schools are centres for cultural education and the formation of cultural identity, and during the initiation process, initiates are taught social norms, customs and values.

As the findings show, a married woman is discouraged from engaging in extramarital relationships, because such promiscuity may bring HIV into the family; similarly, men should respect their wives and avoid coercing them to have sexual intercourse or sleeping with menstruating women, since the risk of contracting HIV is deemed to be very high during that period. Initiation schools for boys and girls are viewed as educational institutions where initiates are educated about courtship, marriage negotiations, their social duty and how to conduct themselves as adults (Mdhluli et al. 2021:02). One of the components perceived to be important regarding such schools is cultural instruction in relation to the roles and responsibilities of what it is to be a ‘man’ or a ‘woman’ (Rathebe 2018:06).

In Vhavenda culture, dress code distinguishes between girls and married women, as a married woman should always portray that she is married. The woman is given a proper name by the husband’s family after marriage to show that they accept her and that others should address her as such. This is deemed to reduce the risk of the woman cheating with other men, which also then reduces the risk of contracting HIV or other STIs. Mapindani (2018:83) concurred, noting that the Tsonga rite of passage for girls and boys is a safe practice, as it protects them against behaving in a manner which might court HIV infection.

Both older girls and boys are mentored to help them become better spouses and parents. To avoid STIs, they are urged not to have sexual intercourse too early and to remain virgins until they marry. If they have marital problems, they know who to turn to for advice. Older girls are advised at *dombani* on how to behave when married. They are also instructed on how to satisfy a man sexually. Dederen and Mokakabye (2018:94) confirmed that the teaching at *dombani* socialises young female novices into normative marital and sexual behaviour. The *domba* ceremony customarily defines womanhood in terms of procreation and motherhood and is primarily aimed at guiding young novices into sexual maturity, marriage and childbirth.

Couples are expected to respect and remain devoted to their spouses. Although men are encouraged to marry multiple women, they are nonetheless required to be faithful to all of the wives and to abstain from becoming promiscuous. Men are also expected to be more responsible than they were prior to being initiated. As Mdhluli et al. (2021:02) confirmed, after initiation a boy becomes a man, and moral expectations are bestowed upon him.

From *thondo*, the initiate is assigned a mediator who advises him on what to do when a problem arises, where to report a pregnancy, what to expect when a woman is menstruating, etc. In the view of the participants, abstaining during menstruation means the couple is protected, as the chance of being infected with HIV through blood during sexual intercourse is high. Nalah, Azlinda and Paramjit (2020:105) are of the view that girls lack guidance and protection, as they are forced to engaged in unprotected sexual intercourse when they are menstruating, putting them at high risk of infected with HIV.

Men are instructed on how to look after a woman and are advised on how to be better fathers. Along with its ability to curb HIV transmission, male circumcision instils discipline and social order amongst young boys. Froneman and Kapp (2017:04) shared the same narratives that at circumcision schools, boys gain discipline and are taught how to behave themselves to become good parents.

The issue of infertility is also dealt with at *thondoni*, where the main goal is to gauge boys’ fertility – if found to be infertile, they are treated by traditional healers in cases where older boys are ready to marry. This counters possible promiscuity where there is little fear of consequences and hence, in the view of the participants, limits the risk of the couple being susceptible to HIV infection. Another view is that as men in north-western Nigeria are not tested and treated for infertility during their traditional initiation schools, women are the ones who suffer the most, as they are blamed for not conceiving. They end up being forced to take traditional medicine so that they can become pregnant, even when they do not have fertility problems (Mohammed-Durosinlorun et al. 2019:06).

Apart from men being assessed, checked and treated for infertility, girls are also checked at all stages of initiation school for virginity to promote abstinence and faithfulness. Those found to still be virgins receive rewards, and those who are already sexually active are encouraged to use condoms to prevent teenage pregnancy and sexually transmitted diseases. In Zimbabwe, virginity testing is performed to curb the spread of HIV and AIDS, to detect incest and abuse and re-instil and promote lost cultural values (Thobejane & Mdhluli 2015).

In Vhavenda culture, children are encouraged to attend *musevhetho* while still young, to ‘tame’ them before they become sexually active. The more people are educated about the dangers of engaging in sexual relations while young, the lower the rate of new HIV cases will be. The stated aim, according to the participants, is to delay sexual activity in youths. The same narrative was shared in the study by Mulea and Choeni (2017:335), which stated that children are advised to abstain from sexual intercourse at *musevhethoni* until they get married to avoid teenage pregnancy and other STIs, including HIV and AIDS.

At *musevhethoni*, girls and boys interact without engaging in sexual intercourse, form friendships, dance, learn social conventions and play games. Girls attend *musevhetho* before starting menstruation – it is similar to a primary school, where they are encouraged to embrace their femininity. Boys at *murunduni* and girls at *musevhethoni* are also taught to adhere to a suitable nutritional diet. They are discouraged from eating high-protein foods (e.g. eggs) to slow early sexual debut, which, if multiple partners are involved, will make them more vulnerable to HIV infection. Sharing the sentiment is the study by Mulea and Choeni (2017:334), which stated that boys and girls are being given rules and guidance on how to behave in all their traditional initiation schools.

Virginity testing is conducted on girls only. Male domination is evident in all stages of initiation – as the study found, most schools are led by men, and it is usually a closed family business. Furthermore, because traditional circumcision is considered sacred, women are not permitted to perform it. Greevy, King and Haffejee (2018:1084) acknowledged gender-based power differentials within such relations, noting that male dominance is often deep-rooted, particularly regarding decisions about condom use during intercourse. While circumcision cannot be practised by women, as Gazimbi (2019:03) confirmed, other ceremonies include: rites of passage, blood sacrifices, cultural markings, the enhancement of male fearlessness and fecundity, preparation for marriage and adult sexuality and the hardening of boys for warfare.

The researchers found that the setting in which initiates are initiated is not clean and hygienic because of the use of temporary plastic and cardboard shelters. One knife is used on all initiates. A contrary practice might be to use a single razor blade per candidate, instead of the traditional knife, which was an important and recent change implemented in Uganda. This change in how traditional initiation ceremonies are conducted, and must consider safety concerns and the risk of HIV transmission (Sabet Sarvestani et al. 2012:01).

It was found that traditional surgeons’ instruments are not properly cleaned and sterilised, with some utilising methylated spirits as disinfectant. Some claim that the knife used has been sanctified by a traditional healer and therefore cannot transmit illness or cause death to initiates. This type of surgical procedure frequently takes place in nonclinical environments, in poor hygienic conditions, where practitioners are not always sufficiently knowledgeable or trained when compared to Western doctors or equipped with proper supplies (Maffioli 2017:05).

Since the incision is made by one traditional surgeon, there is the possibility that he may not wash his hands or change his gloves between initiates when cutting the foreskin. This practice may spread HIV infection, where even one initiate has the disease. As Douglas and Hongoro (2018:598) noted, septicaemia is the main cause of death in traditional circumcision schools. Mdhluli et al. (2021:06) were of the view that circumcision correlates with a decreased danger of HIV infection, urinary tract infection, sexually transmitted diseases and penile carcinoma.

Girls’ clitorises are burned at initiation schools to decrease their libido until they marry. This is done to protect young women from becoming infected with sexually transmitted diseases such as HIV, if one of them is already infected because of early sexual activity. Women are not allowed to seek a divorce and are expected to endure their husband’s behaviour (whether he be faithful or not), and some risk their lives by contracting HIV when opting to stay in a promiscuous marriage. Linganiso and Gwegweni (2016:1031) confirmed that women are more vulnerable to contracting HIV because of their subordinate position in the community, which deprives them of an opportunity to make decisions that affect them during sexual practices. Many circumcised men have multiple partners, as they believe themselves to be immune to HIV. Some even believe using condoms makes them prone to HIV and other STIs, as confirmed by Mutombo, Maina and Jamali (2015:02).

### Limitations of the study

Although this was a comprehensive study, it was undertaken in only one district in the Limpopo province, namely Vhembe. The province consists of four other districts, namely Mopani, Waterberg, Sekhukhune and Capricorn. Participants were mostly traditional healers and community leaders who met the study’s inclusion requirements and thus served as key informants. Community leaders and elders who were involved in initiation schools but refused to participate, as well as individuals who were involved in traditional initiation schools but did not reside in the Vhembe area during the data-collection period, were eliminated. This limitation is inherent, despite the authors’ explanation of the steps taken to ensure the study’s credibility, because the Vhembe district includes people from Vhavenda countries and regions, as well as other areas that have relations with the Vhavenda people of the district. Only three observations and interviews were undertaken to acquire data, which is a drawback because ethnographic studies rely largely on observations. Since some cultural events were considered sacrosanct, the female researchers were not allowed to observe them.

### Recommendations

Young people should be encouraged to attend initiation school, as it will prevent teenage pregnancy and the risk of HIV infection. The activities of the schools must be overseen by the Department of Health (DoH), and doctors who have completed initiation schools must be involved. Risky practices, such as the use of nonsterile knives and burning of young women’s genitals with a wire should be eliminated, and policies and standard operating procedures should be established and implemented properly to benefit both parties (DoH and the House of Traditional Leaders and Healers). If these policies and practices are advertised, schools may be helpful in reducing the number of HIV patients. This can help prevent the spread of HIV if properly monitored and promoted through social media platforms such as Twitter, Facebook, Instagram and TikTok to reach the targeted population, which is primarily young people. Parents and teachers can also be involved by helping young people to understand the practice and accept it. Another suggestion is to involve people who attended traditional initiation schools and are now flourishing, as they can be made the ambassadors for these schools. Youngsters will seek to emulate them and will see that attending initiation school is beneficial to their future, because they will be given rules to guide their sexual behaviour.

## Conclusion

Initiation schools are the backbone of cultural morals and a tool for both girls and boys transitioning to maturity. Rites of passage differ from one culture to the next, but there are known cultural elements in which the practice primarily signifies the transition to adulthood and womanhood and teaches respect, resoluteness, life skills and human philosophy, while protecting initiates from making unwise life choices. Some argue that medical circumcision is better than traditional circumcision. It is also believed that initiates have a higher social position than the uninitiated (*mashuvhuru*), have access to community opportunities, serve as advisors to the king and as previously stated, facilitate their assumption of social responsibility. Overall, these components represent a certain cultural identity and distinctness from other cultural groups. Initiation schools are still an important element of a young man’s passage to manhood. It has been widely noted, however, that such schools have insufficient capacity to assist in delivering HIV prevention information to communities. The formal health and education sectors are the most active in educating youth about HIV and AIDS. Failure to work alongside traditional leaders and elders in educating the younger generation at traditional schools about the danger of prematurely engaging in sexual activity will increase HIV incidence and AIDS-related deaths. This will have a negative impact on the overall goal of raising HIV prevention and treatment awareness.
